# The Use of Anterior-Segment Optical-Coherence Tomography for the Assessment of the Iridocorneal Angle and Its Alterations: Update and Current Evidence

**DOI:** 10.3390/jcm10020231

**Published:** 2021-01-11

**Authors:** Giacinto Triolo, Piero Barboni, Giacomo Savini, Francesco De Gaetano, Gaspare Monaco, Alessandro David, Antonio Scialdone

**Affiliations:** 1 Ophthalmic Institute, ASST Fatebenefratelli-Sacco, 20121 Milan, Italy; francesco.degaetano@asst-fbf-sacco.it (F.D.G.); gaspare.monaco@asst-fbf-sacco.it (G.M.); alessandro.david@asst-fbf-sacco.it (A.D.); antonio.scialdone@asst-fbf-sacco.it (A.S.); 2 San Raffaele Scientific Institute, University Vita-Salute, 20133 Milan, Italy; p.barboni60@gmail.com; 3 Studio Oculistico D’Azeglio, 40123 Bologna, Italy; 4 G.B. Bietti Foundation I.R.C.C.S., 00198 Rome, Italy; giacomo.savini@fondazionebietti.it

**Keywords:** AS-OCT, glaucoma, iridocorneal angle, primary-angle closure, plateau-iris configuration

## Abstract

The introduction of anterior-segment optical-coherence tomography (AS-OCT) has led to improved assessments of the anatomy of the iridocorneal-angle and diagnoses of several mechanisms of angle closure which often result in raised intraocular pressure (IOP). Continuous advancements in AS-OCT technology and software, along with an extensive research in the field, have resulted in a wide range of possible parameters that may be used to diagnose and follow up on patients with this spectrum of diseases. However, the clinical relevance of such variables needs to be explored thoroughly. The aim of the present review is to summarize the current evidence supporting the use of AS-OCT for the diagnosis and follow-up of several iridocorneal-angle and anterior-chamber alterations, focusing on the advantages and downsides of this technology.

## 1. Introduction

Although gonioscopy is currently the gold standard for clinical assessments and diagnoses of angle closure, being also characterized by the unique advantage of allowing a dynamic evaluation of the iridocorneal angle, it is an observer-dependent technique and, thus, is subject to intrinsic intra- and inter-individual variability. Furthermore, it may be challenging and uncomfortable for the patient, as it includes physical contact between the gonioscopic lens and the corneal surface. To overcome these limitations, anterior-segment optical-coherence tomography (AS-OCT) has recently emerged as an objective and noninvasive method to assess the anterior chamber and iridocorneal-angle anatomy.

However, there is no agreement with regard to which parameters extrapolated from the AS-OCT scan are of clinical relevance, nor has it been established whether AS-OCT is comparable to gonioscopy in detecting and monitoring iris and iridocorneal-angle anomalies [1,2,3].

The aim of this review is to summarize the current evidence supporting the use of AS-OCT for the diagnosis and follow-up of several iridocorneal-angle and anterior-chamber alterations.

## 2. Methods

A systematic search was performed across the PubMed, Scopus, and ISI Web of Science databases, using the following queries and/or their combination: ‘anterior segment optical coherence tomography’, ‘anterior chamber optical coherence tomography’, ‘anterior chamber angle’, ‘narrow angle’, ‘iridocorneal angle’, ‘primary angle closure’, ‘primary angle closure glaucoma’, ‘plateau iris syndrome’, ‘plateau iris configuration’, ‘laser peripheral iridotomy’, ‘cataract’, ‘phacoemulsification’. All identified articles underwent initial screening based on title and abstract, which was independently performed by two authors (G.T., G.M.), in order to identify relevant articles. Additionally, the references of identified articles were manually checked to find further potential studies relevant to the present review. All studies available in the literature responding to the above search criteria were then carefully evaluated for quality in terms of clinical relevance, novelty, journal of publication, study design, and sample size, and finally, they were included in the review if both the screening authors agreed that they satisfied the aforementioned requirements. Any disagreement between the two authors performing the search was assessed by consensus, and a third author (P.B.) was consulted if necessary. Articles written in a language different from English were excluded from the review process.

## 3. Relevant AS-OCT Parameters

Several anatomical parameters were proposed for the assessment of the anterior chamber in its entirety, the iridocorneal-angle structures, and the anatomical findings in the iris.

### 3.1. AS-OCT Parameters Concerning the Anterior Chamber in Its Entirety

**Figure 1:**
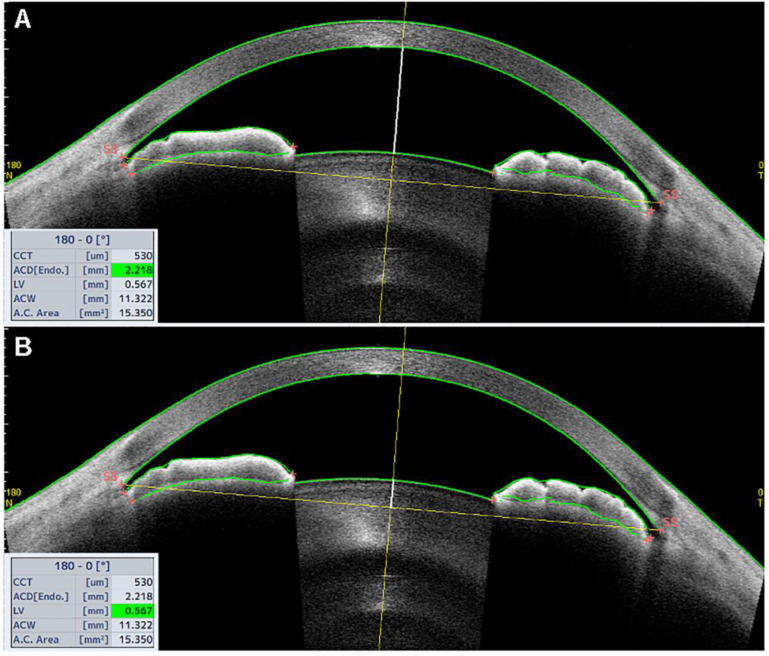
Tomey Casia 2 AS-OCT scan (Nagoya, Japan) of a patient with a narrow angle. (**A**) The white, bold vertical line from the corneal endothelium to the anterior surface of the lens corresponds to the anterior chamber depth (ACD), measuring 2.218 mm in the case reported, as shown in the bottom left table (green highlighted cell). (**B**) The white, bold vertical line from the anterior surface of the lens to the horizontal scleral-spur-to-scleral-spur line corresponds to the lens vault (LV), measuring 0.567 mm in the same patient.

The anterior chamber depth (ACD) is defined as the maximum distance from the corneal endothelium to the anterior surface of the lens, as shown in Figure 1A. In their study, Sng and colleagues reported that the two variables affecting ACD values the most were the lens vault (LV) and the posterior corneal arc length (PCAL) [4]. The LV is defined as the distance from the anterior pole of the lens to the horizontal line connecting the two scleral spurs (SSs), with the SS being defined as the point at which the curvature of the inner surface of the angle wall changes noticeably (often looking as an inward protrusion of the sclera), as shown in Figure 1B [5]. The PCAL is defined as the arc distance of the posterior corneal border between scleral spurs [6]. It has been previously shown that ACD is inversely correlated with age and hyperopic refractive errors [7,8,9], and that decreasing ACD is often associated with primary-angle closure (PAC) [10].

**Figure 2:**
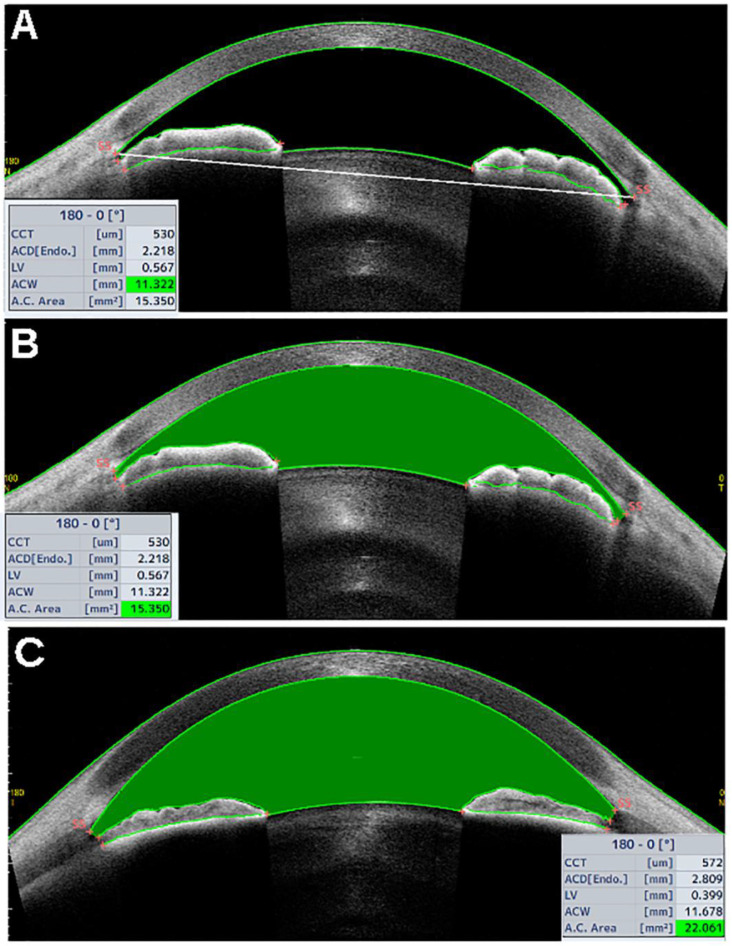
Tomey Casia 2 AS-OCT scans (Nagoya, Japan). (**A**) The white, bold horizontal line corresponding to the scleral-spur-to-scleral-spur line shows the anterior chamber width (ACW) and measures 11.322 mm in this patient with narrow angles. (**B**) The green area, delimited by the corneal endothelium anteriorly, and the anterior surface of the iris and lens posteriorly, corresponds to the anterior chamber area (ACA) and measures 15.35 mm^2^ in the same patient with narrow angles. (**C**) The ACA from a patient with open angles.

Other AS-OCT parameters featuring the AC in its entirety are the anterior chamber width (ACW), area (ACA), and volume (ACV). ACW is defined as the horizontal line corresponding to the scleral-spur-to-scleral-spur distance, as shown in Figure 2A [11]. The ACA is defined as the area delimited by the corneal endothelium anteriorly, and the anterior iris/lens surface posteriorly (Figure 2B,C). The ACA shows inverse correlation to age, while it is directly correlated to the ACD and axial length [7,8,9]. It is unclear whether the ACA has any correlation with the central corneal thickness, as some studies reported inverse correlation between the two parameters [7], whereas others did not [12]. Automated algorithms incorporated into modern AS-OCT devices calculate the ACV based on the ACA acquired through multiple AS-OCT scans to give a three-dimensional measurement of the AC.

### 3.2. AS-OCT Parameters Focusing on Iridocorneal Angle Structures and Iris

**Figure 3:**
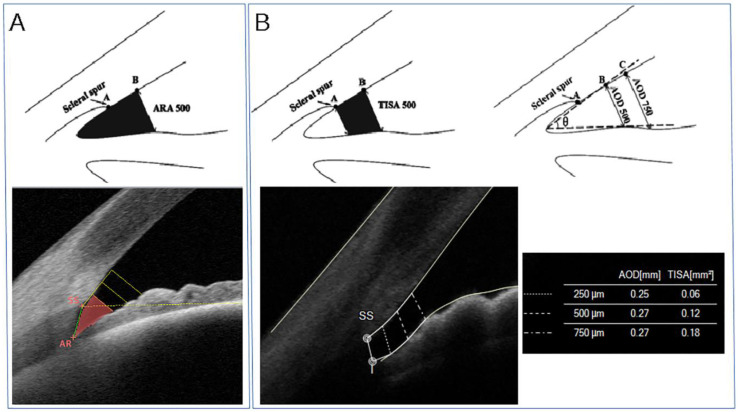
(**A**) **Top panel.** Schematic diagram of the angle-recess area at 500 microns from the scleral spur (ARA500, black area). **Bottom panel.** Tomey Casia 2 AS-OCT scan (Nagoya, Japan) of the angle showing in vivo ARA500 (red area). ARA is defined as the triangular area bordered by the anterior iris surface, corneal endothelium, and a line perpendicular to the endothelium drawn from a point anterior to the scleral spur to the iris surface. (**B**) **Top panel.** Schematic diagram of the trabecular iris-space area at 500 microns from the scleral spur (TISA500, black area, top left panel) and the angle-opening distance at 500 and 750 microns from the scleral spur (AOD500 and AOD750, respectively, top right panel). **Bottom panel.** MS-39 AS-OCT scan (CSO, Florence, Italy) of the iridocorneal angle showing in vivo TISA and AOD. AOD is the perpendicular distance from the iris to the trabecular meshwork, which can be measured at different distances anterior to the scleral spur, such as 250, 500, 750 microns (AOD250, AOD500, AOD750, respectively). TISA, which again can be evaluated at several distances from the scleral spur (SS), is defined as the trapezoidal area with the following boundaries: anteriorly, the AOD; posteriorly, a line perpendicular to the plane of the inner corneoscleral wall drawn from the scleral spur to the opposing iris; superiorly, the inner corneoscleral wall; and inferiorly, the anterior iris surface.

The angle recess area (ARA) is defined as the triangular area bordered by the anterior iris surface, corneal endothelium, and a line perpendicular to the endothelium drawn from a point anterior to the scleral spur (for instance, 750 mm anterior to the SS, in which case it would be ARA750) to the iris surface, as shown in Figure 3A [13].

The angle opening distance (AOD) is the perpendicular distance from the iris to the trabecular meshwork, and it can be measured at different distances anterior to the SS, such as 250, 500, 750 microns (AOD250, AOD500, AOD750, respectively), as shown in Figure 3B.

The trabecular-iris-space area (TISA), which again can be tested and evaluated at several distances from the SS, is defined as the trapezoidal area with the following boundaries: anteriorly, the AOD; posteriorly, a line perpendicular to the plane of the inner corneoscleral wall drawn from the scleral spur to the opposing iris; superiorly, the inner corneoscleral wall; and inferiorly, the anterior iris surface (Figure 3B) [14].

**Figure 4:**
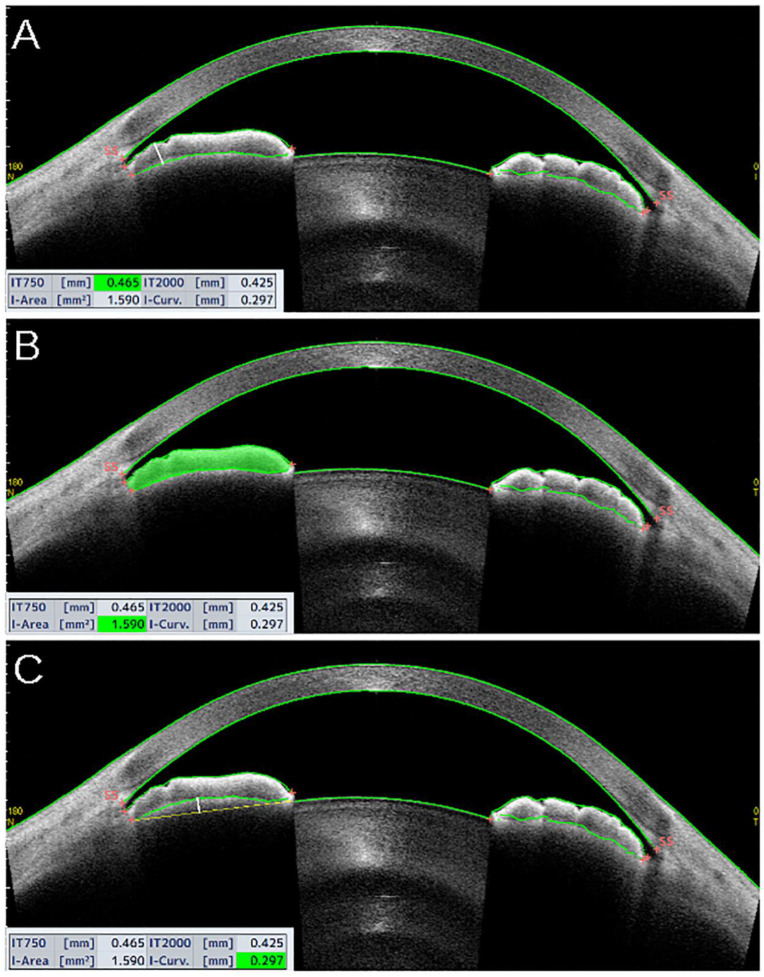
Tomey Casia 2 AS-OCT scans (Nagoya, Japan) showing iris thickness (**A**), area (**B**), and curvature (**C**) from a patient with narrow angles. (**A**) The iris thickness (IT) is the thickness of the iris at different distances from the SS. In the example, IT measured at 750 microns (IT750) from the scleral spur (white, bold vertical line). (**B**) The iris area is defined as the entire cross-sectional area of the iris, from the SS to the pupil. (**C**) The iris curvature is the perpendicular distance from the iris-pigmented epithelium at the point of greatest convexity to a line between the most peripheral and the most central points of the iris-pigmented epithelium (white, bold vertical line).

Some other parameters involve iris measurements. The iris thickness (IT) is the thickness of the iris at different distances from the SS (e.g., IT500, IT750) (Figure 4A). The iris area is defined as the entire cross-sectional area of the iris from the SS to the pupil (Figure 4B). The iris curvature is the perpendicular distance from the iris-pigmented epithelium at the point of greatest convexity to a line between the most peripheral and the most central points of the iris-pigmented epithelium [13,15], as shown in Figure 4C.

### 3.3. Comparison between AS-OCT and Gonioscopy

Nowadays, gonioscopy is still considered the gold-standard test to evaluate the iridocorneal angle. This is due to the fact that it is currently the only test allowing direct anatomical visualization of the angle, including some of its pathological findings (such as peripheral anterior synechiae, signs of appositional angle closure, and pigment dispersion) and the fact that a dynamic test can be performed as well. Therefore, the role of AS-OCT in evaluating iridocorneal angles would be to support and complete gonioscopy, rather than replacing it. However, a direct comparison between gonioscopy and AS-OCT led, in the past, to contrasting results. Initially, Lavanya and colleagues reported a low diagnostic performance of AS-OCT in patients with gonioscopic diagnoses of narrow angles, especially in terms of specificity (with the area under the receiver-operating-characteristic curve being 0.76, sensitivity 88.4%, specificity 62.9%) [3]. Conversely, with the advent of more advanced AS-OCT software, many authors found a relatively good comparison between AS-OCT findings and gonioscopy. Nongipur et al. were among the first to report that the ACW was significantly reduced in patients with gonioscopically-defined narrow angles, compared to subjects with open angles, and in patients with gonioscopic diagnoses of primary-angle closure, compared to patients with narrow angles only [1]. More recently, the same group reported a decreased ACW, ACA, and ACV, an increased LV, and thicker irises in patients with the gonioscopic diagnosis angle closure [2]. Similarly, Wang et al. found that greater iris curvature, area, and thickness, were independently associated with narrow angles defined by gonioscopy [16].

## 4. Clinical Applications of AS-OCT

### 4.1. AS-OCT-Based Diagnosis of Primary-Angle Closure

A smaller ACW, ACA, and ACV, greater iris thickness (IT) and iris area (IArea), and increased LV have been associated with angle closure [8,12,13,15]. Nongipur et al. [2] performed gonioscopy and AS-OCT in 1368 subjects, including 295 (21.6%) who showed gonioscopic angle closure. They found that PAC patients were older, had a smaller ACW, ACA, ACV, a greater LV, and thicker irises compared to patients with open angles (*p* < 0.01 for all). According to these findings, they proposed a classification algorithm based on stepwise logistic regression that used a combination of these six AS-OCT parameters, obtained from a single horizontal scan, able to identify subjects with a gonioscopic PAC >95% of the time. Similarly, Winegarner et al. found that the ACD, ACV, and ACA showed a high diagnostic performance in detecting PAC-disease patients with areas under the receiver-operating-characteristic curve of 0.98, 0.97, and 0.93, respectively [10].

The results from the Chinese-American Eye Study showed that the mean intraocular pressure (IOP) increased as AS-OCT parameters, such as AOD, decreased, and that IOP and AS-OCT findings were inversely correlated in angle-closure patients but not in open-angle ones [17].

Kwon et al. [18] assessed long-term changes occurring to ACD, LV, and AOD in patients with angle closure due to different mechanisms, such as pupillary block (PB), plateau-iris configuration (PIC), thick peripheral iris roll (TPIR), and exaggerated lens vault (ELV). Their study population was followed up for 41 to 54 months, with an average follow-up length of four years. They found that the baseline ACD was shallowest in ELV, followed by PB, TPIR, and PIC, in that order. The PIC group showed significantly wider AOD than the other groups at the baseline. The ACD decreased and the LV increased over time in all groups, especially in the PIC group.

### 4.2. AS-OCT in Plateau-Iris Configuration

**Figure 5:**
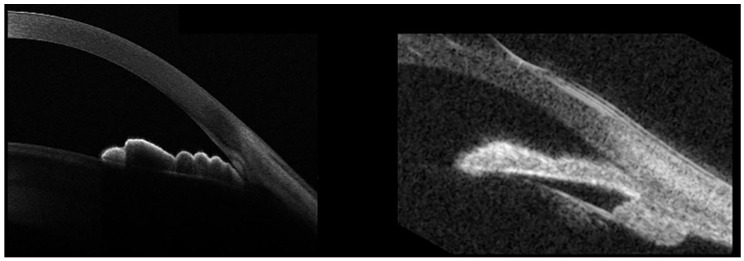
**Left panel.** MS-39 AS-OCT (CSO, Florence, Italy) from a patient with plateau-iris configuration (PIC). **Right panel.** Ultrasound biomicroscopy (UBM) of the same eye. While the AS-OCT scan provides details of the iris surface, iris root, and angle structures, its penetration depth does not make it possible to visualize the ciliary body. Conversely, UBM is characterized by poorer definition of the iris and angle structures, but it also allows the evaluation of the ciliary-body position. This is particularly important in PIC, as it is a condition associated with anterior ciliary-body displacement, which displaces the peripheral iris forward, eventually causing appositional angle closure.

In the plateau-iris configuration (PIC), the iris root points anteriorly on the ciliary body or on the ciliary body itself are displaced more anteriorly than normal, critically narrowing the anterior-chamber recess by displacing the peripheral iris forward (Figure 5). 

PIC is often associated with temporary, appositional angle closure and subsequent raised IOP, eventually leading to glaucoma. It is important to recognize PIC, because it may require additional treatment to the laser-peripheral iridotomy (LPI) usually performed in primary-angle-closure eyes, meaning argon-laser peripheral iridoplasty (ALPI), although the efficacy of this procedure remains debated. Although the diagnosis of PIC is mainly gonioscopic, this technique can be particularly tricky in evaluating the iris root position. In these cases, AS-OCT may offer an objective, noninvasive, and easy tool with which to achieve an accurate diagnosis. While AS-OCT is certainly helpful to evaluate AC and angle details with high-image resolution, it does not allow us to identify an anteriorly-displaced ciliary-body position due to its limited capacity of deep penetration (Figure 5, left panel). Conversely, ultrasound biomicroscopy (UBM) is characterized by poor definition in evaluating the AC and angle-anatomical findings, but it allows good visualization of the ciliary-body position (Figure 5, right). Thus, AS-OCT and UBM provide complimentary information in the evaluation of patients with PIC.

Based on AS-OCT findings, Verma et al. reported that roughly one-third showed incidents of plateau-iris configuration among a group of patients with primary-angle-closure glaucoma [19], suggesting the importance of this imaging technique to evaluate alternative mechanisms to pupillary block in patients with narrow angles. They also identified the iris thickness at 750 microns from the scleral spur (IT750) as the only variable significantly associated with plateau iris.

Interestingly, Crowell et al. reported that lens and pupil parameters evaluated on AS-OCT scans (such as pupil arc, lens vault, and pupil diameter) showed the greatest diagnostic power to discriminate between pupillary block and PIC mechanisms [20].

Although a component of pure pupillary block is often present in patients with PIC, the effects of LPI alone are, at best, controversial. Leaung et al. were among the first in 2005 to report a case of a PAC patient not responding to LPI, although their AS-OCT scans showed PIC [21]. On this patient, ALPI was performed, which proved effective in widening the iridocorneal angles and reducing the IOP.

More recently, Kwon J et al. confirmed that LPI was ineffective in widening the iridocorneal angle in this group of patients [18]. Ramakrishnan et al. reported the effect of ALPI on eyes with plateau-iris configuration, on which LPI had already been revealed to be unsuccessful [22]. They found that on eyes with peripheral anterior synechiae, laser-peripheral iridoplasty can be very effective, not only in lowering IOP and number of IOP-lowering medications, but also in increasing AOD500, TISA500, and the scleral spur angle.

### 4.3. AS-OCT Changes after Laser-Peripheral Iridotomy and Cataract Extraction

In a prospective, observational, randomized, controlled trial of 176 PACS eyes undergoing LPI, this procedure resulted in an increased AOD, TISA, ARA, ACA, and ACV, but not in an increased ACW, ACD, or LV [23]. Similarly, in a prospective interventional case series of 66 PACS eyes, Esfandiari et al. reported that the AOD, ARA, and TISA at 500 mm all increased by 48% to 73% (all *p* < 0.001) after LPI. Conversely, the LV and iris volume did not change significantly [24].

The IMPACT Study, which is a longitudinal, prospective, double-randomized research study, showed similar findings on 39 PAC/PACS eyes [25]. Kwon et al. reported a significant AOD increase in PB and TIPR patients but not in PIC and ELV patients [18]. Moghimi et al. evaluated the effects of LPI on eyes with acute primary-angle closure (APAC) and their fellow eyes [26]. LPI resulted in angle widening with a significant increase in AOD in both eyes with APAC and fellow eyes. Central ACD and ACA significantly increased, and LV decreased, in eyes with APAC, but not in their fellow eyes.

Although relatively short-term changes to AS-OCT parameters are well established after LPI, the same is not the case with long-term changes. For instance, Lee et al. noticed that the AOD, TISA, and ARA significantly increased within two weeks following LPI, but had returned to their pretreatment values at the 18-month follow-up [27]. This finding basically confirmed the previous evidence from the Zhongshan Angle-Closure Prevention Trial, evaluating long-term changes to the iridocorneal angle after LPI in primary-angle-closure suspects [28]. In this randomized, controlled trial, the angle configuration was evaluated using gonioscopy, as well as angle-opening distance at 250, 500, and 750 microns from the scleral spur (AOD250, AOD500, AOD750, respectively), trabecular iris-space area (TISA500, TISA750), and angle-recess area (ARA) measured on AS-OCT images. A significant increase in all AS-OCT parameters was reported two weeks after LPI. Nevertheless, between 2 weeks and 18 months after LPI, a significant decrease in angle width was observed in treated eyes, although this finding was not statistically significant in the first six months. In untreated eyes, the angle width consistently decreased across all follow-up visits after LPI, with a more rapid longitudinal decrease compared to treated eyes.

Yan C et al. used AS-OCT to compare the effects of LPI vs. lens extraction on the anterior-chamber anatomy in PACS eyes [29]. They found that the ACD increased and the LV decreased significantly in the lens-extraction group, but not in the LPI group. The effects of lens extraction on the anatomy of the anterior chamber have already been reported. Huang G et al. showed that ACD and AOD500 increased after cataract surgery, both in patients with preoperatively narrow and open angles, and found a high correlation with IOP-lowering in the former group [30]. Similar findings were confirmed later [31,32], while Nolan et al. reported an increase in the mean AOD500 (88.2%) and TISA750 (94.4%) after cataract extraction in primary-angle-closure glaucoma eyes [33]. More recently, Lee et al. demonstrated that ARA500 and TISA500 increased significantly in healthy eyes and normal-tension glaucoma eyes, with the changes in these parameters being linearly and inversely correlated with postoperative IOP changes [34].

## 5. Discussion

Increasing evidence supports the use of AS-OCT to evaluate the anatomical details of the anterior chamber with a specific focus on the iridocorneal angle. This tool seems particularly helpful in the clinical assessment and follow-up of patients with angle closure and other angle anomalies.

On the one hand, AS-OCT makes it possible to acquire detailed images of the angle anatomy, which, with complimentary gonioscopic examination, may support clinicians in diagnosing iridocorneal-angle alterations and, therefore, eventually help them to determine whether any treatment is needed. On the other hand, AS-OCT scans may provide objective proof of the laser- or surgical-treatment outcomes, and may allow a more accurate follow-up with patients with angle abnormalities.

However, the above-mentioned AS-OCT advantages are counterbalanced by some limitations, which may explain why this technology is still considered a promising and helpful tool but not the gold standard.

First, AS-OCT only provides a static evaluation of the angle anatomy, whereas gonioscopy remains the only diagnostic procedure allowing its dynamic evaluation.

Second, technical limitations may affect, at least partially, the current clinical usefulness of AS-OCT. With the future advent of more sophisticated technology, the AS-OCT may provide more precise and detailed information about the anterior-segment anatomy, and this will hopefully improve the capacity of identifying iridocorneal-angle structures and detecting related anomalies.

Finally, although an increasing number of AS-OCT parameters may help us to better understand the anterior-chamber anatomy and its relationship to angle-closure disease, this may generate some confusion in evaluations of AS-OCT scans. Furthermore, it needs to be taken into account that at least some of the AS-OCT parameters may not show clinical significance and may go out of fashion in the coming years.

## Data Availability

Because of the review nature of the present manuscript, no original data were collected.
